# Neuronal and hormonal perturbations in postural tachycardia syndrome

**DOI:** 10.3389/fphys.2014.00220

**Published:** 2014-06-16

**Authors:** Philip L. Mar, Satish R. Raj

**Affiliations:** Departments of Medicine and Pharmacology, Vanderbilt University School of Medicine Nashville, TN, USA

**Keywords:** postural tachycardia syndrome, aldosterone, angiotensin II, blood volume, hyperadrenergic activity, Autonomic Nervous System, neuropathy, orthostatic intolerance

## Abstract

The Postural Tachycardia Syndrome (POTS) is the most common disorder seen in autonomic clinics. Cardinal hemodynamic feature of this chronic and debilitating disorder of orthostatic tolerance is an exaggerated orthostatic tachycardia (≥30 bpm increase in HR with standing) in the absence of orthostatic hypotension. There are multiple pathophysiological mechanisms that underlie POTS. Some patients with POTS have evidence of elevated sympathoneural tone. This hyperadrenergic state is likely a driver of the excessive orthostatic tachycardia. Another common pathophysiological mechanism in POTS is a hypovolemic state. Many POTS patients with a hypovolemic state have been found to have a perturbed renin-angiotensin-aldosterone profile. These include inappropriately low plasma renin activity and aldosterone levels with resultant inadequate renal sodium retention. Some POTS patients have also been found to have elevated plasma angiotensin II (Ang-II) levels, with some studies suggesting problems with decreased angiotensin converting enzyme 2 activity and decreased Ang-II degradation. An understanding of these pathophysiological mechanisms in POTS may lead to more rational treatment approaches that derive from these pathophysiological mechanisms.

## Introduction

Postural Tachycardia Syndrome (POTS) is a debilitating syndrome that is characterized by symptoms of presyncope when assuming an upright position. This syndrome is the most common disorder seen in autonomic specialty clinics and affects 500,000–3,000,000 individuals in the United States (Robertson, 1999). Young women are disproportionately affected, with nearly 80–85% of cases occurring in women and most of childbearing age (Garland et al., 2007). The cardinal hemodynamic feature of this syndrome is an increase in heart rate (HR) by ≥30 bpm on assuming an upright position within 10 min in the absence of orthostatic hypotension (a drop in systolic blood pressure (BP) >20 mmHg or a drop in diastolic BP >10 mmHg) (Freeman et al., 2011; Raj, 2013). In addition, symptoms of orthostatic intolerance (palpitations, light-headedness, chest discomfort, or dyspnea) must accompany this orthostatic tachycardia, improve with recumbency and persist for at least 6 months. Symptoms must also occur in the absence of conditions that cause orthostatic tachycardia, such as prolonged bedrest, use of medications that impair autonomic regulation (vasodilators, diuretics, antidepressants, or anxiolytic agents), or chronic debilitating disorders that cause tachycardia (such as dehydration, anemia, or hyperthyroidism) (Raj, 2013).

Neurohormonal dysregulation has been identified in a number of patients with POTS. We will discuss the data supporting this implication and review the neurologic and hormonal aspects of POTS as it pertains to the pathophysiology of this condition.

## Pathophysiology of POTS (Table 1)

**Figure 1:**
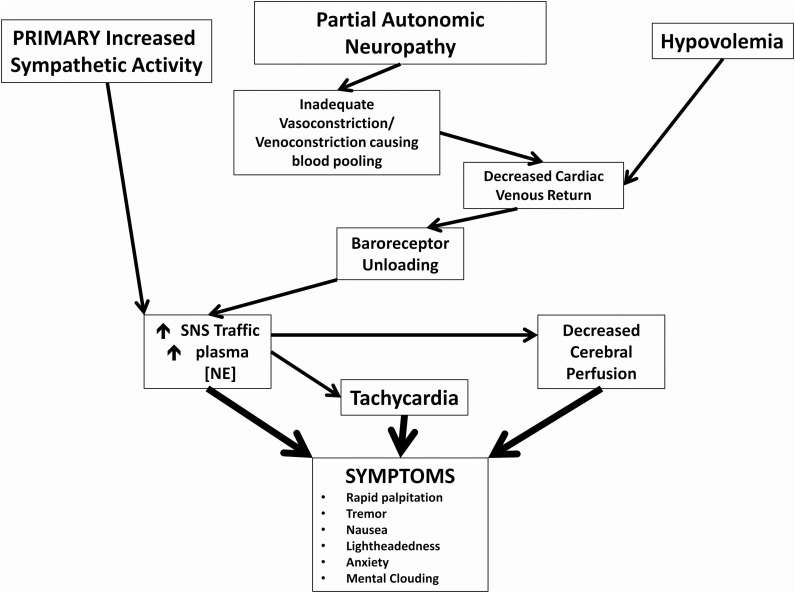
**Pathophysiological mechanisms of postural tachycardia syndrome.** Cartoon representation of how 3 major neuronal and hormonal abnormalities and their immediate effects may cause symptoms commonly associated with POTS.

POTS is a heterogeneous syndrome with several different pathophysiological mechanisms that can result in the typical POTS presentation, and a few more common ones are highlighted in this manuscript (Figure 1) (Raj, 2013). Neuropathic POTS is a condition with a partial neuropathy where there is preferential denervation of sympathetic nerves in the lower limbs that may account for local/regional blood flow abnormalities including venous pooling (Jacob et al., 2000; Stewart et al., 2003). A state of hypovolemia also exists in the majority of POTS patients. This phenomenon of low blood and plasma volumes in the presence of inappropriately low levels of renin and aldosterone in POTS patients has been referred to as the “renin-aldosterone paradox” (Raj et al., 2005a). This hypovolemic state can lead to decreased venous return, and contributes to presyncopal symptoms in addition to reflex tachycardia. Though many POTS patients have elevated plasma norepinephrine secondary to partial autonomic neuropathy or hypovolemia, central hyperadrenergic POTS is a variant of POTS where individuals have high levels of upright plasma norepinephrine in the absence of these two findings (Raj et al., 2005a; Mustafa et al., 2011; Raj, 2013). A few patients in one family have a specific genetic abnormality linked to a single point mutation in the norepinephrine transporter with subsequent diminished clearance of norepinephrine (Shannon et al., 2000). This hyperadrenergic state is thought to drive the orthostatic tachycardia in these patients (Figure 1). The different pathophysiological mechanisms are not mutually exclusive, and POTS patients will often have an overlap of features from a number of these aforementioned variants.

**Table 1:** **Some pathophysiological mechanisms of POTS and related treatments**.

**Major pathophysiological mechanisms of POTS**	**Pathophysiology**	**Treatments**	**Mechanism of therapy**
Partial autonomic neuropathy	Partial autonomic neuropathy in lower extremities	Midodrine (Jacob et al., 1997; Hoeldtke et al., 2006; Lai et al., 2009; Ross et al., 2014)	An alpha-1 agonist that increases peripheral vasoconstriction
	Abnormal splanchnic blood flow and pooling	Octreotide (Hoeldtke and Davis, 1991; Hoeldtke et al., 2006)	A somatostatin analog that decreases splanchnic blood flow
Perturbed renin-angiotensin aldosterone system and hypovolemia	Inappropriately low levels of renin and/or aldosterone	Exercise (Fu et al., 2011)	Precise mechanism unclear, but increases renin:aldosterone ratio
	Low blood and/or plasma volume	Exercise (Fu et al., 2011)	Increases plasma volume
		Fludrocortisone (Freitas et al., 2000)	A mineralocorticoid that increases sodium and water retention
		Erythropoietin (Hoeldtke et al., 1995; Kanjwal et al., 2012)	A hormone that increases blood volume
		Saline Infusions (Jacob et al., 1997)	Acutely increases plasma volume
		DDAVP (Coffin et al., 2012)	An ADH analog that increases intravascular volume
Hyperadrenergic State	Increased secretion and clearance of norepinephrine	Propranolol (Raj et al., 2009; Fu et al., 2011);	A non-selective beta-blocker that impairs sympathetic activation
		Pyridostigmine (Raj et al., 2005b; Singer et al., 2006; Kanjwal et al., 2011)	An acetylcholinesterase inhibitor that increases parasympathetic activity and slows heart rate

### Neuropathic POTS (partial autonomic neuropathy)

In 1988, Streeten et al. first published a report regarding the pathophysiology of POTS. In 34 patients with symptoms of orthostatic intolerance, 10 patients exhibited orthostatic increases in HR >30 bpm. In these 10 individuals, radioisotopic measurements of orthostatic pooling of blood in the calf was significantly greater compared to healthy subjects (*P* < 0.01), which was suggestive of at least partial autonomic neuropathy related to a portion of the autonomic system (Streeten et al., 1988).

#### Abnormal blood flow abnormalities in the lower extremity

Stewart and Weldon confirmed increases in orthostatic leg volume and venous blood flow consistent with excessive pooling in the lower extremities in a pediatric POTS population using strain-gauge measurements (Stewart and Weldon, 2000). They further dichotomized POTS patients into two groups based on lower extremity venous pressure (VP) > 20 mmHg (high-VP POTS) or ≤20 mmHg (low-VP POTS) and found defective vasoconstriction in both groups as evidenced by significantly more blood flow in the calves during orthostasis compared to healthy subjects. While supine, high-VP POTS group had normal arterial resistance but lower blood flow in the lower extremities compared to healthy subjects, and the low-VP POTS group had less arterial resistance and higher blood flow in the lower extremities compared to healthy subjects (Stewart and Weldon, 2001). High-VP POTS patients, also referred to as “low-flow” POTS patients (LFP), had inappropriate vasodilation during orthostasis instead of the vasoconstriction that was seen in healthy subjects and “high-flow” POTS (HFP) patients. The excessive blood pooling in the lower extremities and increased orthostatic leg volume is due to a defect in arteriolar vasoconstriction and not an abnormality of venous capacitance (Stewart, 2002; Stewart et al., 2003). These studies indicate there is an abnormal vascular response in the extremities that predisposes POTS patients to venous pooling secondary to arteriolar dysregulation.

#### Abnormal regional blood volume regulation

Both Doppler ultrasound and segmental impedance plethysmography (Diedrich and Biaggioni, 2004) have indicated that there is abnormal blood flow and pooling in the splanchnic circulation of many POTS patients (Tani et al., 2000; Stewart and Montgomery, 2004). Both “low-flow” and “high-flow” patients had significantly greater decreases greater decreases in thoracic blood flow versus healthy subjects during orthostasis (*P* < 0.025 and *P* < 0.004 respectively). In addition, “low-flow” POTS patients had increased splanchnic blood flow compared to healthy subjects (*P* < 0.01) with upright position (Stewart et al., 2006b). “High-flow” POTS patients had increased pooling in the pelvis and legs versus healthy subjects (*P* < 0.05 and *P* < 0.025 respectively) (Stewart and Montgomery, 2004).

#### Partial autonomic neuropathy

An explanation for this adverse blood pooling is a partial autonomic neuropathy. Schondorf and Low initially found evidence of generalized autonomic neuropathy in patients with POTS (Schondorf and Low, 1993). Later, Jacob et al. showed that there was a defect in sympathetic nervous system innervations of the lower extremity in POTS patients (Jacob et al., 2000). They demonstrated that norepinephrine spillover, or the norepinephrine that was released at sympathetic synapses and “spilled over” into the venous circulation, was considerably impaired. This defect in norepinephrine spillover was predominantly in the lower extremities. Using a cold pressor test, a nitroprusside infusion, and tyramine infusion, they demonstrated that there was decreased norepinephrine spillover in POTS patients compared to healthy subjects in each of the 3 tests (*P* = 0.02, *P* = 0.01, and *P* = 0.04 respectively). In contrast, systemic and upper extremity norepinephrine spillovers were unchanged in POTS patients compared to healthy subjects. Taken together, these data suggest that some patients in POTS have inadequate sympathetic tone to the lower extremities leading to diminished vasoconstriction and venoconstriction. At this point, it is unclear if this is due to denervation and/or impaired norepinephrine release at the synaptic cleft of these peripheral sites. Nevertheless, what results is diminished venous return and decreased stroke volume, with a secondary increase in central sympathetic nerve traffic that results in excessive orthostatic tachycardia.

### Renin-angiotensin aldosterone system (RAAS) and hypovolemia in POTS

#### Hypovolemia and the “Renin-Aldosterone Paradox”

In more recent years, hormonal research as it relates to the pathophysiology of POTS has converged on the renin-angiotensin aldosterone system. Low blood volume (red cell volume and plasma volume) has been demonstrated in multiple studies in POTS patients (Jacob et al., 1997; Raj et al., 2005a; Stewart et al., 2006a; Fu et al., 2010). Raj et al. showed that a cohort of POTS patients specifically have inappropriately normal levels of plasma renin activity (PRA) and paradoxically lower levels of aldosterone (*P* = 0.017) despite their hypovolemia when compared to healthy subjects (Raj et al., 2005a). PRA activity was similar even after 30 min of standing. In addition, the aldosterone: renin ratio was considerably lower in the POTS group versus the healthy subjects (*P* = 0.047). Stewart et al. showed that the “low-flow” group of POTS patients specifically had significantly lower levels of PRA when compared to healthy subjects (*P* < 0.05) (Stewart et al., 2006a). Fu et al. in a study of 10 premenopausal women with POTS, found that PRA significantly rose after 2 hours of standing compared to healthy subjects, while aldosterone did not change significantly (Fu et al., 2010). They also confirmed a reduced aldosterone:renin ratio in POTS patients.

#### Angiotensin II (Ang-II)

Subsequent work has also shown that plasma Ang-II levels in some POTS patients are elevated when compared to healthy subjects (Stewart et al., 2006a; Mustafa et al., 2011). Ang-II is the major effector of the RAAS axis, causing systemic vasoconstriction, raising BP, and is critical for maintaining fluid balance homeostasis through aldosterone secretion (Zhuo and Li, 2011). Elevations of Ang-II in POTS patients are on the order of 2-3 times higher than healthy subjects (Stewart et al., 2006a; Mustafa et al., 2011). The absence of hypertension in these patients is therefore perplexing. Mustafa et al. infused a standard dose of Ang-II into POTS patients and healthy subjects (Mustafa et al., 2012). They found that POTS patients have a blunted systemic vascular and hypertensive response to Ang-II versus healthy subjects. Importantly, Ang-II infusion induced a similar amount of aldosterone production in POTS patients as it did in healthy subjects. Renal blood flow and vascular response was also unchanged between POTS patients and healthy subjects (Mustafa et al., 2012). The full implications of increased plasma Ang-II in POTS patients is still unclear.

#### Pleiotropic effects of the AT_1_ receptor (AT_1_R)

Ang-II exerts most of its physiologic effects though AT_1_R (De Gasparo et al., 2000). This G-protein coupled receptor elicits multiple cellular responses via coupling to G_*q*_ proteins. While most of its action are rapid and attributed to G-protein meditated secondary messengers, there is increasing evidence that the internalization of Ang-II by AT_1_R can produce long-lasting genomic and gene transcriptional effects via continued activation of intracellular targets (Zhuo and Li, 2011). AT_1_R is a very pleiotropic receptor, found in a variety of different tissues, including adrenal, neuronal, cardiac, renal, and vascular smooth muscle cells (De Gasparo et al., 2000). Some of its actions are consistent throughout the body, while some will vary based on the location of the receptor. For example, while it generally elicits hypertensive responses via its actions on vascular smooth muscle cells (VSMC) in most tissue beds, it has hyperproliferative effects only on VSMC in the cerebral vasculature, and not on VSMC located peripherally (Hunyady and Catt, 2006). Additionally AT_1_R also plays an important role in the central nervous system where Ang-II acts like a neurotransmitter, modulating BP, salt intake, thirst mechanisms, and other neuroendocrine processes (De Gasparo et al., 2000). The pleiotropic nature of this receptor, along with its multiple pathways of activation, contributes to the versatility of Ang-II. This concept is relevant to the discussion of POTS physiology because a response will vary based on the specific tissue being studied.

#### Microvascular nitric oxide dysfunction

Medow et al. found that there was defective cutaneous vasodilation of the microvasculature mediated by nitric oxide with local heating in POTS patients versus healthy subjects (Medow et al., 2005). Neuronal nitrous oxide synthase (nNOS), and not endothelial nitrous oxide synthase (eNOS), was determined to be responsible for causing this phenomenon (Stewart et al., 2007). Ang-II plays an important role in this defect of microvascular vasodilation because the administration of an angiotensin type 1 receptor (AT1R) blocker, losartan, reverses this defect in POTS patients (Stewart et al., 2008). In summary, the skin blood flow defect present in POTS patients can be simulated in healthy subjects by infusing a nNOS inhibitor, and can be reversed in POTS patients by infusing an Ang-II antagonist. These skin findings may play a role in the dependent acrocyanosis seen in many POTS patients. There are not yet any studies that have assessed whether these skin findings have systemic vascular implications.

#### Angiotensin converting enzyme 2 (ACE2)

ACE2 is a monocarboxypeptidase that metabolizes Ang-II, an octapeptide, into Angiotensin 1-7 [Ang(1-7)]. Ang(1-7) has vasodilatory properties and has actions that generally oppose that of Ang-II (Chappell, 2007; Zhuo and Li, 2011). Ang(1-7) also plays a role in skin microvascular dysregulation (Stewart et al., 2009; Mustafa et al., 2012).

Stewart et al., showed that with local infusion of losartan and a NOS inhibitor, cutaneous vasodilation due to local heating is reduced in healthy subjects to the level of POTS patients (Stewart et al., 2009). However, even in the presence of losartan and a NOS inhibitor, the infusion of Ang-II can reverse this reduction in cutaneous vasodilation only in healthy subjects, whereas the lack of vasodilation persists in POTS patients. The source of this recovery in vasodilation in healthy subjects after Ang-II infusion is thought to be peripheral conversion of Ang-II into Ang(1-7) as addition of an ACE2 inhibitor will sabotage this vasodilation in healthy subjects. Similarly, in POTS patients, even in the presence of NOS and losartan, infusion of Ang(1-7) can restore normal cutaneous vasodilation. Thus, the source of this dysregulation is thought to represent a deficiency of ACE2 in the skin of POTS patients.

Mustafa et al. also showed that this deficiency in ACE2 extends into the systemic circulation by measuring the ratio of Ang(1-7) to Ang-II and used it as a surrogate for functional ACE2 activity (Mustafa et al., 2011). In the presence of elevated systemic levels of Ang-II with comparable levels of Ang1-7, POTS patients had a significantly lower ratio of Ang(1-7):Ang-II when compared to healthy subjects (*P* = 0.038).

#### Further investigations

The source of ACE2 dysfunction in POTS patients is still unclear. A specific genetic mutation could be the cause. Alternatively, ACE2 dysfunction could be a downstream manifestation resulting from POTS. As most POTS patients are intolerant of physical activity, ACE2 dysfunction could be a product of general deconditioning. Prior research has shown that diet, at least in the short term, does not affect level of ACE2 activity (Mustafa et al., 2011).

Another possibility is that the measured Ang-II might not really be Ang-II. Most prior studies that have quantified Ang-II have employed assays that were not sensitive enough to distinguish Ang-II from angiotensin 3 or angiotensin 4 since they typically employed radioimmunoassays that targeted the peptides common to the C-terminus (Stewart et al., 2006a; Mustafa et al., 2011). It is possible that some of the measured “Ang-II” is actually angiotensin 3 or angiotensin 4.

The exact mechanism of how a defect in ACE2 might trigger the clinical manifestations of POTS is also still poorly understood. While there may be a deficiency in peripheral and cutaneous vasodilation secondary to an ACE2 defect, it is not clear how that produces orthostatic tachycardia and presyncopal symptoms.

In addition to the “renin-aldosterone paradox” with the lack of aldosterone response in the presence of hypovolemia, the high Ang-II levels and low ACE2 activity remains to be explained.

### Hyperadrenergic POTS

Previous studies in normal healthy subjects have demonstrated normal supine plasma norepinephrine levels to be around 200 pg/mL (Jacob et al., 1998). With standing, upright norepinephrine levels plateau below 600 pg/mL after 7.5 min. Supine plasma epinephrine levels are around 25 pg/mL and increase with standing up to 70 pg/mL. Numerous studies have documented comparable supine levels of norepinephrine and epinephrine between POTS and healthy subjects, but elevated upright norepinephrine (Jacob et al., 2000; Raj et al., 2005a; Mustafa et al., 2011). Tachycardia is the most salient manifestation of this hyperadrenergic state. Another manifestation of increased SNA during orthostasis in POTS patients includes possibly increasing coherence and blunting cerebral autoregulation leading to decreased cerebral blood flow (Ocon et al., 2009). However, it should be noted that there are several studies in the literature with inconsistent findings related to cerebral blood flow during orthostasis in POTS patients (Jordan et al., 1998; Schondorf et al., 2005; Ocon et al., 2009).

Using muscle sympathetic nerve activity (MSNA) as measured by microneurography, sympathetic nervous activity (SNA) in POTS patients has been shown to differ from that of healthy subjects at rest (Furlan et al., 1998), during orthostasis (Muenter et al., 2005), or induced hypotension (Bonyhay and Freeman, 2004). Although data available is conflicting regarding resting SNA activity in POTS patients, POTS patients have an exaggerated SNA response compared to healthy subjects during orthostatic and hypotensive challenge (Bonyhay and Freeman, 2004; Muenter et al., 2005).

One etiology behind this exaggerated SNA response is attributed to norepinephrine transporter (NET) dysfunction. Administration of a NET inhibitor, reboxetine, to healthy subjects has been shown to produce a POTS phenotype with increase of HR in response to head-up tilt testing by greater than 30 bpm (Schroeder et al., 2002). NET inhibition is thought to increase norepinephrine concentrations acting on postsynaptic adrenoreceptors, which drives the tachycardia in POTS. The disproportionate response in HR can be explained by the heart's essential reliance on the NET (Esler et al., 1991).

This hyperadrenergic state can be “secondary” such as in response to hypovolemia, or “primary” such as one related to a genetic mutation. Shannon et al. demonstrated that a specific genetic mutation can cause POTS. A specific missense mutation in the exon of the norepinephrine transporter gene (*SLC6A2*) produced an Ala457Pro mutation in the norepinephrine transporter causing 98% loss of function (Shannon et al., 2000). The mutation was isolated in a 33 year old woman with a 20 year history of orthostatic intolerance as well as her identical twin sister. Plasma supine norepinephrine levels were normal for both patients (269 pg/mL and 199 pg/mL), but both patients had upright plasma norepinephrine levels exceeding 900 pg/mL, and 1 patient became hypertensive with standing. Although most patients with hyperadrenergic POTS do not have this genome mutation, epigenetic modification at this gene locus which decreases expression of the NET protein has also been associated with POTS patients. Bayles et al. demonstrated that the promoter of the *SCL6A2* gene was especially sensitive to histone modifications that downregulated expression of this protein (Bayles et al., 2012). The data here demonstrates inhibition of the norepinephrine clearance transporters, which is a mechanism of many psychotropic medications, predisposes individuals to elevated levels of upright norepinephrine and can produce a typical POTS phenotype.

There is also some data that the parasympathetic system may contribute to the tachycardia in POTS. Furlan et al. reported that high frequency R-R interval variability (0.15–0.4 Hz), a marker of parasympathetic activity, was reduced in POTS patients compared to healthy subjects during passive orthostatism (Furlan et al., 1998). Thus, decreased cardiovagal activation due to reduced parasympathetic nervous system activity may contribute to POTS.

## Treatment approaches: Addressing neurohormonal imbalances (Table 1)

### Partial autonomic neuropathy—alpha-1 agonists

Alpha-1 agonists have been used in POTS patients to restore the lack of adrenergic vasoconstriction due to partial autonomic neuropathy in the lower extremities. Phenylephrine infusions have previously been shown to improve HR and enhance peripheral vasoconstriction in POTS patients. However, phenylephrine infusion also increased BP in these patients (Stewart et al., 2002). Jacob et al. have shown that midodrine, an orally active alpha-1 agonist at 5-10 mg doses, is very effective at reducing the orthostatic tachycardia at 1 and 2 hours after administration. In addition it had very minimal effects on the BP and also reduced supine HR (Jacob et al., 1997). The addition of a beta-blocker in addition to midodrine enhanced the therapeutic efficacy when compared to midodrine alone (Lai et al., 2009). More recently, it was shown that “high-flow” POTS patients were much more responsive to the treatment of midodrine than “low-flow” POTS patients (Ross et al., 2014).

Octreotide is a somatostatin analog that causes vasoconstriction in the splanchnic vascular bed. It has been shown to significantly reduce orthostatic tachycardia in POTS patients to a similar extent that midodrine does (Hoeldtke and Davis, 1991; Hoeldtke et al., 2006). Standing times were increased when midodrine was added to octreotide treatment in POTS patients, but neither midodrine nor octreotide alone significantly increased standing times (Hoeldtke et al., 2006).

### Hypovolemia and renin-angiotensin-aldosterone paradox—volume loading

Fludrocortisone is a potent fluorinated aldosterone agonist that causes significant sodium and water retention (Thorn et al., 1955). It has been widely and successfully used for the treatment neurogenic orthostatic hypotension (Freeman, 2008; Freeman et al., 2011). Fludrocortisone is also considered a first-line treatment for POTS and has been shown to improve symptoms significantly (Freitas et al., 2000; Raj, 2013). Before starting blood volume expansion agents, medications that directly antagonize aldosterone, such as spironolactone or drosperinone (which is found in certain oral contraceptives) should be stopped first.

Desmopressin (DDAVP) is an orally available synthetic analog of arginine vasopressin. In a study of 30 POTS patients, the short-term administration of DDAVP orally reduced the degree of orthostatic tachycardia compared to placebo significantly (*P* < 0.001). In addition, patients reported significant improvements in overall symptoms, and specifically symptoms related to vision, tremulousness, and palpitations (Coffin et al., 2012).

Erythropoietin has also been used to treat the decreased blood volume in POTS patients by artificially increasing erythropoiesis. Hoeldtke et al. initially found no improvement in reducing orthostatic tachycardia in a small study involving 8 patients with orthostatic intolerance after 6–12 weeks of erythropoietin treatment. Furthermore, supine as well as standing BP were elevated (Hoeldtke et al., 1995). In a larger study of 39 POTS patients refractory to conventional treatment, erythropoietin administration garnered no improvement in orthostatic tachycardia. However, a significant proportion (~80%) of these patients reported subjective improvement of their symptoms (Kanjwal et al., 2012).

The use of volume loading with intravenous saline is effective at acutely relieving the symptoms of POTS. Jacob et al. showed that the infusion of 1 liter of normal saline over a time period of 1 h was effective in significantly reducing orthostatic tachycardia at 1 h upon the completion of the infusion (Jacob et al., 1997). The results of this are short-lived. Some patients have pursued chronic IV saline infusions, but this is not widely advised due to concerns about access complications.

Fu et al. conducted a trial before-after exercise training to 3 months of an exercise regimen consisting of 4 sessions per week, each lasting 30–45 min (*n* = 19) (Fu et al., 2011). The 3 month exercise program was able to increase the aldosterone:renin ratios well as the plasma volumes and blood volumes in these POTS patients. Other benefits included a reduction in orthostatic tachycardia (*P* < 0.01), and significantly improved quality of life as assessed by the SF-36.Exercise is the only intervention that has been shown to improve the aldosterone:renin ratio and increase plasma and blood volumes over the long term.

### Hyperadrenergic state—heart rate control

Attempts to manage the hyperadrenergic state in these individuals center around HR control. The use of beta-blockade to decrease HR has been met with conflicting data. Masuki et al. had previously shown that POTS patients had reduced stroke volumes and required a faster upright HR to maintain cardiac output (Masuki et al., 2007). Stewart et al. showed that acute esmolol infusions did not significantly reduce orthostatic HR (Stewart et al., 2002). However, Raj et al. showed that the use of propranolol at a 20 mg dose acutely and significantly reduced orthostatic tachycardia compared to placebo (*P* = 0.010) up to 4 h after administration (Raj et al., 2009). Low dose propranolol also improved symptoms significantly during this time. Fu et al. found that long-acting once daily propranolol did not improve quality of life at 1 month (Fu et al., 2011).

Pyridostigmine, an acetylcholinesterase inhibitor, has also been used to manage orthostatic tachycardia (Raj, 2013). The putative mechanism behind using an acetylcholinesterase inhibitor is to increase parasympathetic tone by enhancing cholinergic activity at both the ganglionic nicotinic and the postganglionic muscarinic acetylcholine receptors (Raj et al., 2005b). In a study of 17 patients, standing HR was significantly reduced at 2 and 4 h after pyridostigmine administration vs. placebo (*P* < 0.001 for both times). Although pyridostigmine also acts to enhance sympathetic transduction at nicotinic ganglia, BP were unaffected. Symptoms also were significantly improved 4 h after administration of pyridostigmine. Subsequent studies by Singer et al. and Kanjwal et al. have confirmed the long-term benefits of using pyridostigmine (Singer et al., 2006; Kanjwal et al., 2011).

Given that a defective norepinephrine transporter has been implicated in causing hyperadrenergic POTS, medications that inhibit norepinephrine reuptake worsen tachycardia in POTS patients (Shannon et al., 2000). Vincent et al. showed that in normal healthy individuals, higher doses of duloxetine, a serotonin-norepinephrine reuptake inhibitor (SNRI), caused healthy volunteers to develop a POTS-like phenotype with increased orthostatic tachycardia (Vincent et al., 2004). Green et al. have recently reported that atomoxetine increased standing HR in POTS patients and that it acutely worsened symptom burden in 2/3 of the POTS patients (Green et al., 2013). In addition, selective-serotonin reuptake inhibitors (SSRI) have also been shown to inhibit norepinephrine reuptake, (Shores et al., 2001) and recent reports show that the acute administration of the SSRI, sertraline, acutely worsened symptoms in POTS patients vs. placebo (Mar et al., 2014). These findings must be balanced against their potential long-term benefits in terms of managing anxiety from a chronic illness and coping with POTS. Data for these long-term effects are currently lacking.

## Conclusions

Over the past 20 years, considerable research has taken place in an effort to understand and explain this enigmatic syndrome. Several neural and hormonal observations have been made in regard to the pathophysiology of POTS. POTS patients have variously been shown to have a partial neuropathic state with impaired lower extremity sympathetic innervations, abnormal venous pooling, a hypovolemic state with inadequate RAAS upregulation, cutaneous blood flow dysregulation, and also increased plasma Ang-II levels. In recent years, pathophysiology research for POTS has shifted toward work on the RAAS, and considerable emphasis has been placed on the Ang-II/ACE2/Ang(1-7) axis (Stewart et al., 2008, 2009; Mustafa et al., 2011, 2012).

As the etiology of POTS is not completely understood, it is unclear if the Ang-II/ACE2/Ang(1-7) axis is the unifying pathologic mechanism that drives the pathophysiology for all the manifestations of POTS that do not as of yet have a crystal-clear explanation. Alternatively, the manifestations for each variant may simply be one of several but typical downstream responses to a defective Ang-II/ACE2/Ang(1-7) axis or another unifying pathologic mechanism. If so, then the exact steps that link these manifestations to that unifying pathologic mechanism will need to be elucidated.

## Research funding

Supported in part by NIH grants R01 HL102387, P01 HL56693, and UL1 RR024975 (Clinical and Translational Science Award).

### Conflict of interest statement

National Institutes of Health grants R01 HL102387, P01 HL56693, and UL1 RR024975 (Clinical and Translational Science Award). The authors declare that the research was conducted in the absence of any commercial or financial relationships that could be construed as a potential conflict of interest.
