# Corrigendum: Neuronal and hormonal perturbations in postural tachycardia syndrome

**DOI:** 10.3389/fphys.2019.00978

**Published:** 2019-07-30

**Authors:** Philip L. Mar, Satish R. Raj

**Affiliations:** Departments of Medicine and Pharmacology, Vanderbilt University School of Medicine, Nashville, TN, United States

**Keywords:** postural tachycardia syndrome, aldosterone, angiotensin II, blood volume, hyperadrenergic activity, Autonomic Nervous System, neuropathy, orthostatic intolerance

There was an error in the original article. A reference to low-frequency R-R interval variability was made instead of high frequency R-R interval variability. Furthermore, it was incorrectly stated that decreased cardiovagal activation and its contribution to POTS is unclear, while it should have stated that it may contribute to POTS.

A correction has been made to the section **Pathophysiology of POTS (Table 1)**, subsection **Hyperadrenergic POTS**, paragraph five:

“There is also some data that the parasympathetic system may contribute to the tachycardia in POTS. Furlan et al. reported that high frequency R-R interval variability (0.15–0.4 Hz), a marker of parasympathetic activity, was reduced in POTS patients compared to healthy subjects during passive orthostatism (Furlan et al., 1998). Thus, decreased cardiovagal activation due to reduced parasympathetic nervous system activity may contribute to POTS.”

The authors apologize for this error and state that this does not change the scientific conclusions of the article in any way. The original article has been updated.

## References

[B1] Furlan R. Jacob G. Snell M. Robertson D. Porta A. Harris P.. (1998). Chronic orthostatic intolerance: a disorder with discordant cardiac and vascular sympathetic control. Circulation 98, 2154–2159. 9815870 10.1161/01.cir.98.20.2154

